# Strengths and Weaknesses of Children Witnessing Relatives with Amyotrophic Lateral Sclerosis

**DOI:** 10.3390/children10010140

**Published:** 2023-01-11

**Authors:** Ines Testoni, Lorenza Palazzo, Lucia Ronconi, Tamara Macelloni, Vincenzo Calvo

**Affiliations:** 1 Department of Philosophy, Sociology, Pedagogy and Applied psychology (FISPPA), University of Padova, 35139 Padova, Italy; 2 Emili Sagol Creative Arts Therapies Research Center, University of Haifa, Haifa 3498838, Israel; 3 Multifunctional Centre of Psychology, University of Padova, 35139 Padova, Italy

**Keywords:** reflective functioning, amyotrophic lateral sclerosis, representation of death, death education

## Abstract

Research on minors who have a close family member with amyotrophic lateral sclerosis (ALS) is scarce. This study aims to analyze the relationships between reflective function and wellbeing among such children, considering their reflective function, representations of death, and behavioral problems with the following instruments: Reflective Functioning Questionnaire, Testoni Death Representation Scale for Children, Positive and Negative Affect Schedule for Children, and Strengths and Difficulties Questionnaire. Participants were 248 minors divided into the target group (38 children–16 females, 22 males–7–18 years old (M = 11.61, SD = 2.97)) and the control group (210 students–120 females, 90 males 9–14 years old (M = 11.17, SD = 1.33)). Results showed that the target group exhibited more negative affect and hyperactivity. However, they also showed less uncertainty in their mental states. The opportunity to support these minors is discussed.

## 1. Introduction

ALS is a progressive, neurodegenerative disease that affects motor neurons and the nerve and spinal cord cells that enable voluntary muscle movements [1]. The disease progresses more or less rapidly causes important changes in lifestyle and family comportment are required to manage the patient’s condition [2]. This reorganization and reciprocal adaptation reflects the patient’s growing dependence on their caregivers, and numerous studies have shown that this condition, together with pain, undermines the quality of life of patients and even their families [3]. 

The primary caregiver assumes the tasks related to patient care and these roles involve increasing levels of economic, emotional, physical, and nursing care, communication with health care staff, and assistance with treatment-related decision-making. Although patients feel positive about the caregiver’s quality of care [4], the caregiver often reports feeling burdened, stressed, and even depressed, probably due to the progression of the disease, which causes an increased sense of isolation and prevents family members from elaborating their feelings about the situation [5]. Despite these difficulties, there are few empirical studies on the effects of ALS on minors who live with an ALS patient. One study by Calvo et al. [6] showed that they are at greater risk for anxiety, suffering, depression, and psychosocial difficulties. The more externally visible the illness is, the more significant these reactions in children will be, and they will also be accompanied by concern and denial of the situation [7,8,9].

Minors in this type of family face several sources of uncertainty caused by ambiguous conditions. The first one is due to the confusion of boundaries (boundary ambiguity) between parental and filial roles, as children may assume some caretaking responsibilities on behalf of their parents, further obfuscating their mourning [10]. Indeed, they intervene in disease management and personal care tasks, medication, emotional support, and housework, often whilst lacking in information and skills [11]. Another area of ambiguity is related to the aspect of communication, due to the unfeasibility to speak and manifest emotions with the facial expressions. In particular, the risk of misunderstanding due to the patient’s inability to express himself normally is important. A further difficulty is related to the experience of “ambiguous loss”, which entails a form of grief that is difficult to consolidate, given that the loved one is not dead, but radically changed [12]. This phenomenon comes from seeing a person suddenly fragile and highly demanding, as in the case of neurodegenerative diseases that cause the physical presence of the patient but the disappearance of his/her mental abilities (e.g., dementia) or the reverse, the presence of mental abilities but total paralysis (e.g., ALS) [13]. Ambiguous loss may cause significant emotional distress for family members, including loss of hope, depression, anxiety, relational conflicts, and somatization. Minors, in particular, may feel lonely or insecure, and they may not be able to understand the sense of loss they are experiencing [14].

Situations of ambiguity can generate problems in the ability to understand oneself and others. In particular, the ability to understand the mental states of others and differentiate them from one’s own is called the reflective function (RF), which is acquired in the first years of life and then progressively advances over time. RF has a social and relational value because it allows people to respond adaptively to social interactions [15] and to regulate affectivity [16]. It consists of an intra-personal dimension, composed of self-awareness and understanding, and an interpersonal dimension, that is, the ability to see others as psychological entities with independent thoughts, emotions, and needs [17,18]. Moreover, it is related to the theory of mind, that is the ability to infer one’s own and others’ mental states and to distinguish them [19]. The RF depends on the child’s relationship with their attachment figures [18,20].

Loss caused by death may be potentially upsetting, and hurtful events interact with reflective function by affecting the ability and desire to empathize with the other person to understand his or her mental states, and making critical situations more difficult to understand [16,21,22]. The suffering caused by death is related to the ‘representation of death’, that is the attitudes of individuals toward the idea of existing beyond death (death is a passage to another form of existence) or not (death is absolute annihilation) [23]. Literature showed that those who approach the vision of death as ‘annihilation’ tend to have less hope for the future and a loss of resilience, where the representation of death as a ‘passage’ constitutes a protective factor against anxiety and fear of death and brings greater confidence, security, hope and serenity than the thought of death [24,25]. These factors are decisive and protective for resilience.

Given this background, it is hypothesized that minors who experience a parent with ALS disease face on the one hand the problem of ambiguity and loss, and on the other hand the fear that their parent will die: both of these conditions might affect their wellbeing and reflective function.

## 2. Materials and Methods

### 2.1. Aims

The first aim was to adapt the instruments not previously validated in the Italian language for the survey and to analyze the correlations among the study variables. Second, we wanted to observe whether there were any differences in reflective function and representations of death in two groups of minors: the study group with minors with experience of a close family member with ALS and the control group with no such experience.

### 2.2. Participants

There were 248 minors involved in this study, and they were divided into the target group (38 children, of whom 16 were females) aged between 7 and 18 years old (M = 11.61, SD = 2.97) and the control group composed of 210 students, most of whom were females (120 females, 90 males) aged between 9 and 14 years old (M = 11.17, SD = 1.33), the latter of which was divided in two age groups: from 9- to 10-year-olds (72 students–39 females, 33 males) and from 11- to 14-year-olds (138 students–81 females, 57 males). In the study group, most of the children (16, 42.11%) attended primary school, 9 (23.7%) were in middle school, and the remaining were at high school (13, 34.2%) All the participants were Italian; 36 were of the Catholic religion, and 2 of the Islamic religion. 

Regarding the level of kinship with the family member with ALS, the participants in the study group were divided as follows: 6 children (15.79%) had a grandfather or grandmother who was ill, 2 (5.26%) had a cohabiting aunt, and most of them (30, 78.95%) had either parent. Almost all of the relatives with ALS (*n* = 24) were at an advanced stage of the disease: 10 (42%) were quadriplegic, 9 (37.5%) had PEG (Percutaneous Endoscopic Gastrostomy) and were tracheostomized, 7 (30%) had impaired walking and used a medical walker, 8 (33.3%) showed loss of speech, 5 (21%) were paraplegic, 1 (4.17%) had mild behavioral changes, and 1 (4.17%) had frontotemporal dementia. No significant differences between the two groups were found for gender distribution and mean age. In the control group, the participants were all Italians and, regarding their school level, 72 attended primary school (34.29%) and 138 were from middle school (65.71%).

Informed consent was obtained by all participants’ parents before administering all the questionnaires and the interview. The study was approved by the Ethical Committee for Psychological Research at the authors’ institution (reference: C3DD8C5FCE1C26C7E80954B4EC34DC16).

### 2.3. Method

The project started with a collaboration with an Italian non-profit association that offers support on different levels to ALS patients and their families. Families already afferent to the association, who had children or grandchildren of patients with ALS, were told about the project and offered to participate on a voluntary basis. It was specified to the families that participation would be free of charge and the choice to participate or not would not affect the additional services provided by the association.

The participants of the study group completed the questionnaires with a psychologist trained on the research project who illustrated the study to the families and guided the minors in completing the questionnaires. A snowball sampling was used for the control group starting with some schools with which the researchers already had contact and expanded to other schools based on the willingness of the principals and parents of the pupils to participate. The administration of questionnaires for the control group participants took place within classrooms during school hours under the supervision of a researcher. Both groups of participants took an average of 40 min to complete the questionnaires.

### 2.4. Instruments

The questionnaires included the following instruments: the Reflective Functioning Questionnaire (RFQ), the Testoni Death Representation Scale (TDRS-C), the Positive and Negative Affect Schedule for Children (PANAS-C), and the Strengths and Difficulties Questionnaire (SDQ).

The RFQ is a 7-point Likert scale questionnaire that is used to evaluate the psychological processes underlying one’s ability to mentalize, i.e., the ability to think and reflect on one’s own and others’ behavior. We used the 8-item, reduced version of this instrument [26]. It is composed of two scales. The first measures one’s uncertainty about mental states (RFQ-U) to check ‘hypomentalization’, i.e., the inability to create a mental model of oneself or others. The second measures one’s certainty about mental states (RFQ-C) to check ‘hypermentalization’, i.e., the excess of mentalization, which corresponds to the development of very complex models of mental states [27,28]. High levels of either variety of mentalization are considered negative, as they indicate one’s difficulty to understand the ‘opacity’ of a person’s mental state. Our validation of the Italian version of the questionnaire showed good internal consistency (Cronbach’s α = 0.77 for the Certainty scale and α = 0.75 for the Uncertainty scale). The test also reliably predicted the presence or absence of a psychopathological disorder, which was a significant factor distinguishing the two groups involved in the validation [26,28].

The TDRS-C is a 7-point Likert scale self-report questionnaire adapted from the adult version [23] for use with children and pre-adolescents. It evaluates one’s ontological representation of death based on a continuum wherein one pole represents death as annihilation and the other represents death as a passage. In the version for children and pre-adolescents, participants are asked to respond to four statements by choosing a number from 1 to 5, wherein 1 corresponds to ‘I totally disagree’ and 5 corresponds to ‘I totally agree’. The adult version of the questionnaire has good internal consistency (α = 0.86).

The PANAS-C [29,30] is a 27-item self-report scale that measures children’s Positive and Negative Affect. It was adapted from the adult version, PANAS [31]. In it, participants are asked to rate how often they have experienced a number of feelings over the past few weeks using a 5-point Likert scale ranging from 1 (very slightly or not at all) to 5 (extremely). PANAS-C has shown adequate internal consistency and moderate convergent and discriminant validity compared to other, traditional self-report measures for child depression and anxiety [30].

The SDQ [32,33] is a 25-item questionnaire designed to measure the psychological adjustment of children and adolescents. It includes five scales, each of which assesses a different behavioral and emotional dimension (Emotional Symptoms, Conduct Problems, Hyperactivity—Inattention, Peer Problems, and Prosocial Behaviour) on a 3-point Likert scale wherein 1 means ‘Not true’, 2 means ‘Somewhat true’, and 3 means ‘Certainly true’. Higher scores on the Prosocial Behaviour subscale denote a child’s strengths, whereas higher scores on the other four subscales indicate a child’s emotional or behavioral difficulties. The SDQ questionnaire has been used extensively in both clinical and nonclinical settings. The reliability of the Italian self-report version seems to be less satisfying for younger children than for older children, but it has shown adequate psychometric characteristics that are acceptable and coherent with results from older children [32].

### 2.5. Statistical Analysis

First, we analyzed the reliability of the RFQ and TDRS-C scales’ factor structure by calculating Cronbach’s alpha coefficients for each scale. If the result of the alpha coefficient was between 0.60 and 0.70 or higher, then the scale was deemed to be reliable [34]. Next, we used the *R package lavaan* [35] to carry out Confirmatory Factor Analysis (CFA) with the maximum likelihood estimation method. Hu and Bentler [36] suggested that the Tucker–Lewis Index (TLI = NNFI) and Comparative Fit Index (CFI) should be equal to or greater than 0.95 to determine adequate goodness of fit. Meanwhile, we used Akaike’s Information Criterion (AIC) [37] values to determine the best model (by definition, the model with the smallest value). Netemeyer et al. [38] suggested the following statistical thresholds: chi-square (χ2) *p*-value higher than 0.05; Goodness of Fit Index (GFI) greater than or equal to 0.90; and Root Mean Square Error of Approximation (RMSEA) smaller than or equal to 0.08.

Second, we examined the distribution of all composite scores to check the normality. Asymmetry and kurtosis were evaluated according to Kim’s guidelines [39]: we considered a cut-off of 2 for skewness and 7 for kurtosis, values higher than the cut-offs provide evidence of non-normality. No variable exceeded the cut-offs for skewness and kurtosis, indicating that the normality assumption was satisfied. Pearson correlation coefficient was used for correlations between all the measures considered in the study. When examining the correlations, we used the following classification of effect sizes: values from 0.10 to 0.25 indicate a small effect, those from 0.25 to 0.40 a medium effect, and those over 0.40 a large effect [40]. We used an ANOVA 2 × 2 factorial design to analyze sex and age group differences in the control group. Finally, we used independent samples *t*-tests to analyze the differences between the target group and the control group. We used Cohen’s *d* [40] to estimate effect sizes.

## 3. Results

### 3.1. First Aim: Validation of Instruments Used in the Study

Table 1 shows the reliability results for the two factors of the RFQ. We see that the factor Certainty about mental states exhibited adequate reliability (α = 0.64), whereas the factor Uncertainty about mental states exhibited low reliability (α = 0.49). All but one of the item-total correlations for RFQ_C scored 0.30 or over, whereas only two item-total correlations for RFQ_U scored 0.30 or over. The mean scores were very similar (M = 0.93, SD = 0.68 for RFQ_C; M = 0.83, SD = 0.58 for RFQ_U), indicating low levels for both factors.

Table 2 shows the reliability results for the total score of TDRS-C. We see that this measure exhibited good reliability (α = 0.83) and had a large effect size for all item-total correlations.

The PANAS-C exhibited very good reliability for both Positive Affect (α = 0.87) and Negative Affect (α = 0.89). The SDQ exhibited good reliability for Emotional symptoms (α = 0.67), Hyperactivity–inattention (α = 0.63), Peer problems (α = 0.60), Total difficulties (α = 0.79), Internalizing problems (α = 0.70), and Externalizing problems (α = 0.73), and low reliability for Prosocial behavior (α = 0.59) and Conduct problems (α = 0.53).

To test the construct validity of the RFQ, we created and compared several CFA models. First, we tested the unidimensionality of each dimension of the RFQ. Based on the RMSEA, CFI, and GFI, we confirmed that both the Certainty about mental states factor and Uncertainty about mental states factor were unidimensional (see Table 3). Afterward, we conducted two total CFA models—a one-factor model and a two-factors model—to ensure that the underlying components were mutually exclusive. We compared these models with the AIC model and found that the most adequate structure of RFQ seemed to be the two-factors model. Although the correlation between the two factors was strong (r = −0.70), the two factors did not completely overlap.

For the TDRS-C validation, we constructed only one CFA model: a one-factor model. All indices confirmed adequate goodness of fit (Table 3).

Table 4 shows the correlations between measures. Almost all correlations were statistically significant, with only a few exceptions. In particular, representations of death as annihilation did not correlate with RFQ factors. Positive Affect did not correlate with RFQ factors, but Negative Affect correlated negatively with Certainty about mental states (r = −0.23, *p* = 0.001) and positively with Uncertainty about mental states (r = 0.30, *p* < 0.001). Much like negative affect, all SDQ measures showed negative correlations with Certainty about mental states (correlations were from −0.20 to −0.46, except for Peer problems and Internalizing problems) and positive correlations with Uncertainty about mental states (correlation coefficients ranged from 0.26 to 0.44). Moreover, correlations involving Externalizing problems were stronger than correlations involving Internalizing problems. Representations of death as total annihilation correlated negatively with Positive Affect (r = −0.22, *p* = 0.002) and positively with Externalizing problems (r = 0.21, *p* = 0.002). Finally, Positive Affect correlated negatively with all SDQ measures (correlations were from −0.20 to −0.37) and positively with Prosocial behavior (r = 0.22, *p* = 0.001). On the contrary, Negative Affect showed positive and stronger correlations with all SDQ measures (correlations were from 0.29 to 0.51).

Table 5 shows the ANOVA 2 *×* 2 (Sex x Age groups) results for all measures. With regard to the RFQ, there was a significant interaction between sex and age groups for the Certainty about mental states factor, but not for the Uncertainty about mental states factor. In particular, we see a significant decline in Certainty about mental states from 9–10 years to 11–14 years in females (*t =* −2.74, *p* = 0.007), and no significant variation in males (*t =* 0.90, *p* = 0.370). With regard to the TDRS-C, sex was a significant main effect, and there was a significant interaction between sex and age groups. Males were more likely to view death as total annihilation than females (M = 2.68, SD = 1.19 and M = 2.38, SD = 1.04, respectively), but this difference was statistically significant only at 9–10 years (*t =* 2.90, *p* = 0.004) and not at 11–14 years (*t =* 0.42, *p* = 0.676). Further, there was a significant increase in perceptions of death as total annihilation between age groups for females (*t =* 2.68, *p* = 0.008) but not for males (*t =* −0.41, *p* = 0.683). In addition, age was a significant main effect for Positive Affect, as 9- to 10-year-old students had higher scores than 11- to 14-year-old students, and age and sex were significant main effects for Negative Affect, while also demonstrating a significant interaction. In particular, females scored higher in Negative Affect than males (M = 32.53, SD = 12.33 and M = 26.93, SD = 8.69, respectively), and 11- to 14-year-old students scored higher in Negative Affect than 9- to 10-year-old students (M = 31.58, SD = 11.80 and M = 27.35, SD = 9.57, respectively), but gender differences were significant for 11- to 14-year-old students and not for 9- to 10-year-old students (*t =* 4.45, *p* < 0.001 and *t =* 0.10 *p* = 0.931, respectively), and age differences were significant for females and not for males (*t =* 3.62, *p* < 0.001 and *t =* 0.19, *p* = 0.850, respectively). Finally, with regard to the SDQ, sex was the only significant main effect for Emotional problems, Internalizing problems, Hyperactivity, and Prosocial behavior. In particular, females scored higher than males on Emotional problems (M = 4.25, SD = 2.34 and M = 2.66, SD = 2.37, respectively) and the total of Internalizing problems (M = 6.08, SD = 3.25 and M = 4.51, SD = 3.63, respectively), but also on the strength point of Prosocial behavior (M = 7.90, SD = 1.70 and M = 7.23, SD = 1.62, respectively). Instead, males scored higher than females on Hyperactivity (M = 3.90, SD = 2.18 and M = 3.38, SD = 2.23, respectively).

### 3.2. Second Aim: Examination of the Experiences of Children Living under the Specter of a Family Member with ALS

Children in the target group exhibited significantly higher negative affect than their peers in the control group (Table 6). They also exhibited significantly more problems with Hyperactivity–inattention and significantly less Uncertainty about mental states.

To investigate any differences according to the age of the participants, the target group was divided into two age groups: 21 children from 7 to 12 years and 17 from 13 years upwards. No significant differences were observed in the sample based on age group. Concerning gender, however, a single significant difference was observed with regard to the subscale of the Prosocial Behaviors of the SDQ which is greater in females (*t =* 3.50 *p* = 0.001; M = 8.01 SD = 1.68 vs. M = 7.27 SD = 1.63). This result is in line with previous uses of the SDQ on the Italian sample [32,41].

## 4. Discussion

This study aimed to adapt tools for the investigation and analyze the correlations between the study variables; moreover, it aimed to investigate any differences between minors experiencing the ALS of a parent or grandparent and minors without experience of ALS (control group) and to observe whether and how it impacts on their wellbeing. Compared to the Italian adaptation of RFQ and TDRS-C for the research objectives, seeing their results with PANAS-C and SDQ, acceptable results were obtained. The use of RFQ with children and adolescents yielded lower coefficients of validity than the Italian version for adults [28], but the correlation between the two factors of the scale was similar. The reliability of the scale was lower when it was used with younger children than when it was used with adolescents aged from 12 to 18 years [42]. As for the children’s certainty and uncertainty about the RF, our results are in line with the literature [42]; that is, uncertainty about one’s mental states correlates positively with internalizing and externalizing behavioral problems, which were measured in this study with the SDQ; meanwhile, certainty about one’s mental states correlates negatively with negative dimensions of behavior or emotions and positively with cognitive empathy and mindfulness. In addition, in line with results from studies that used Ensink’s [43] Child Reflective Functioning Scale to measure children’s RF, the RFQ scale adapted by the present study did not present significant sex effects in the results. As with Ensink’s study [43], our RFQ adaptation showed a positive correlation between uncertainty about mental states and externalizing behaviors.

Regarding the representation of death, the first validation of the TDRS with Italian adults presented an average score that did not express any clear preference for death as a passage or death as annihilation [23]. In the present study, we confirmed the results of other studies that used the TDRS with adolescents [44,45]; that is, the children’s total average scores suggested a representation of death as annihilation. No sex differences were observed in the initial validation of the TDRS [23], but some were observed in the present study. In fact, males scored more highly on death as annihilation than females did, though females showed a significant increase in this perspective with age (even these, however, were lower than similarly aged males’ scores). This finding is in line with what we observed in a previous study, wherein we used this scale with a larger sample of university students [46]. However, this higher score in the representation of death as annihilation contrasts with the findings of other studies conducted with children between 10 and 12 years old: children of this age tended to give fewer responses that referred to death as the definitive end or the cessation of biological processes and more responses referring to a biological and spiritual afterlife, focusing on what remains after death and supernatural aspects [47,48]. Because the representation of death as annihilation is associated with anxiety and fear of death, our results indicate that there is a social need to implement educational reflection on death, life, and transcendental dimensions. Higher levels of spirituality and a mature concept of death improve children’s emotional well-being and enable them to elaborate on the idea of finitude [49,50,51,52]. Given that children’s scores on the TDRS-C scale correlate negatively with the positive dimensions of affectivity (PANAS-C) and prosociality (SDQ) and positively with the negative behavioral dimensions of SDQ, we hypothesize that school-based death education programs, which promote discussion of the existential issues related to spirituality and transcendental dimensions, may promote self-awareness, emotional resilience, compassion, and empathy among children [23,53,54,55].

With respect to the SDQ, females scored higher in prosocial and emotional behaviors than males, in line with the existing literature [32,41,56]. Moreover, males scored higher in hyperactivity than females, as they did in Tobia and colleagues’ study [56]. In previous uses of the scale, the self-report version of the SDQ demonstrated less internal reliability than the questionnaire completed by parents; however, it is still a valid tool. Nonetheless, Muris and colleagues [57] assert that, although the self-report values from subjects aged 8–10 years are comparable to those from subjects aged 11 years or more, the reliability of the scales appears to be less satisfactory for the younger age group, and they advise researchers and clinicians to administer the instrument more cautiously to children under 11 years of age. With regard to positive and negative affect, as measured in the PANAS-C, the present study found significant sex differences, in line with the Italian validation of the scale and other international literature. Females in the older age group scored higher in negative affect than similarly aged males, while those in the younger age group scored higher in positive affect than similarly aged males [29,58].

Once the instruments were validated, we compared the results from the control group with those from the target group. One aim of the study was the detection of differences in RF and representation of death among the two groups of participants. With regard to RF and the relative ability to mentalize, the target group yielded lower scores on the RFQ—both in certainty and uncertainty about mental states—and a significantly lower level of uncertainty than the control group. Some studies show that young people’s knowledge about the disease may have a beneficial effect on the caregiver: when minors are more aware of the disease, they are more likely to support and help the caregiver [59]. Informing children about ALS could help them to cope with the frustrations and difficulties associated with changes in daily life and the need for personal sacrifice; on the other hand, not being informed about the condition of one’s parent can damage the relationship with him/her and lead to a lack of trust, implying feelings of isolation and marginalization [60,61]. The lower uncertainty in the target group is probably due to the greater maturity of these minors, who, compared to their peers in the control group, had to develop reflective skills earlier than is typical in order to manage their everyday experiences, as observed in the previous literature reported above [62]. Being aware of the disease situation increases resilience and maturity: they strive to maintain their agency, autonomy, and decision making by seeking strategies to cope with changes in daily life and sociality, despite the demands of care and its emotional impact [61,63,64]. However, the intrapersonal RF seems to be lacking in some children. Potential psychological interventions should take this finding into account, as the progression of stressful experiences can have a negative impact on minors’ ability and desire to understand and verbalize their mental and emotional states, or even to identify those of others. This makes traumatic situations more difficult to understand, reducing the child’s resilience and increasing their vulnerability [20].

With respect to the representation of death, which was measured by the TDRS-C, the target group scored in the middle of the two visions of death, but slightly closer to a representation of death as annihilation. On the one hand, it would be important to delve into this aspect and investigate the spiritual dimension of families with ALS, since, in situations of illness, spirituality turns out to be a protective factor in dealing with death anxiety and also in the search for meaning for what is happening and what will happen to one’s loved one [65,66,67]. On the other hand, this result suggests the need to investigate the experiences of these families and minors by investigating the presence of sincere dialogue regarding death and the level of awareness of these minors, in order to investigate the possible link between a representation of death such as annihilation and the lack of dialogue on this topic in the family. Being able to talk openly about one’s emotions about death, in fact, reduces the fear and anxiety of death and is a topic that even minors, from an early age, need to address.

With regard to affect which was measured through the PANAS-C, the target group scored significantly higher in negative affect than the control group. This finding is consistent with that of previous studies [6,63,64], wherein affected children were more likely than their peers to internalize their emotional reactions, experience difficulties at school, and fret about the condition of their sick family member. In the SDQ, children in the target group also scored higher in hyperactivity–inattention than the control group. However, it is important to note that this attribute may not be entirely negative: hyperactivity–inattention is sometimes a defensive strategy that children adopt to alleviate their anxiety and to escape from their stressful family situation [63]. Certainly, this strategy is only adaptive insofar as it does not prevent the child’s recognition of their own emotions or their ability to genuinely relate to their family members. This is a very real risk, these children have been given important responsibilities at an early age, changing their role from care receiver to caregiver who supports both parents while denying their own needs. Taking care of others certainly demonstrates healthy psychosocial development, but, over time, the child may also feel overwhelmed by these demands and responsibilities [59]. In addition, the limitations that this variety of care imposes on social experiences may hinder the development of the child’s identity, producing an image of the self that is based on caretaking responsibilities [9,68].

## 5. Conclusions and Limitations

Concerning the first aim, the positive correlation between uncertainty about one’s mental states with internalizing and externalizing behavioral problems was confirmed; moreover, the negative correlation between certainty about one’s mental states and negative dimensions of behavior or emotions and the positive correlation of it with cognitive empathy and mindfulness was found, as in the existing literature. Comparison of the groups responding to the second objective showed that the target group scored lower in the reflective function, in both of its components, and this may be explained by the fact that the experience to which these minors are exposed caused them to develop reflective skills earlier and in a more mature form. The target group, as expected, showed more negative affectivity and hyperactivity–disattention.

Both groups did not come significantly closer to either representation of death, but both scored closer to annihilation. 

This study showed that there is need for interventions of support for minors and also interventions that prepare families to face the theme of illness and death with patience and competence, as well as for those that enable reflection on spiritual dimensions. Thinking of the loved one as freed from illness, in a further reality where it is possible to stay in touch, may help children to overcome the difficulties and pain that this topic entails. One limit of the present study that could be overcome in future research concerns the low reliability (Cronbach’s alpha) of the RFQ_U scale. Future studies should also find more participants in the 9- to 10-year age range. Unfortunately, cultural avoidance and denial of death causes significant resistance to this kind of study, making it difficult to find schools that are willing to participate in the research.

A further limitation is the age difference between the control group and the target group and also the uneven distribution of participants with respect to gender differences.

Furthermore, the control group was constituted by taking whole classes willing to participate in the project. For these reasons, it was not possible to find a group of the same age as the target one.

Finally, a final limitation of the present study is the different health conditions of family members with ALS (i.e., those who have PEG and tracheostomy who have pro-longed their lives compared to those who decided not to have PEG and tracheostomy), which might in some way impact on the concept of death of the children involved. In future studies, it might be useful to investigate this aspect and these differences in the concept of death through qualitative methodology as well and further compare any differences in children with parents with diseases other than ALS to observe differences in the two constructs investigated as well. Since the development of a psychological and reflective self, endowed with the ability to think about others in terms of mental states, is also the result of adequate parental RF [18,22,69,70], future research on this topic could use the RFQ to clarify the relationship between the parent’s and child’s RF.

## Figures and Tables

**Table 1:** Reliability of Reflective Functioning Questionnaire (RFQ) ^1^.

Factors	Item	Statements	Coding	Corrected Item-Total Correlation
RFQ_C	1	People’s thoughts are a mystery to me	(1 = 3) (2 = 2) (3 = 1) (4 thru 7 = 0)	0.19
α = 0.64 M = 0.93; SD = 0.68	2	I don’t always know why I do what I do	(1 = 3) (2 = 2) (3 = 1) (4 thru 7 = 0)	0.36
	3	When I get angry I say things without really knowing why I am	(1 = 3) (2 = 2) (3 = 1) (4 thru 7 = 0)	0.52
	4	When I get angry I say things that I later regret	(1 = 3) (2 = 2) (3 = 1) (4 thru 7 = 0)	0.29
	5	If I feel insecure I can behave in ways that put others’ backs up	(1 = 3) (2 = 2) (3 = 1) (4 thru 7 = 0)	0.33
	6	Sometimes I do things without really knowing why	(1 = 3) (2 = 2) (3 = 1) (4 thru 7 = 0)	0.55
RFQ_U	2	I don’t always know why I do what I do	(1 thru 4 = 0) (5 = 1) (6 = 2) (7 = 3)	0.33
α = 0.49 M = 0.83 SD = 0.58	4	When I get angry I say things that I later regret	(1 thru 4 = 0) (5 = 1) (6 = 2) (7 = 3)	0.19
	5	If I feel insecure I can behave in ways that put others’ backs up	(1 thru 4 = 0) (5 = 1) (6 = 2) (7 = 3)	0.24
	6	Sometimes I do things without really knowing why	(1 thru 4 = 0) (5 = 1) (6 = 2) (7 = 3)	0.37
	7	I always know what I feel	(1 = 3) (2 = 2) (3 = 1) (4 thru 7 = 0)	0.24
	8	Strong feelings often cloud my thinking	(1 thru 4 = 0) (5 = 1) (6 = 2) (7 = 3)	0.16

^1^ In the analysis, only the students who completed all items of RFQ (*n* = 205) are included.

**Table 2:** Reliability of Testoni Death Representation Scale for Children (TDRS-C) ^1^.

Factors	Items	Statements (English Version)	Coding	Corrected Item-Total Correlation
Total TDRS-C α = 0.83 M = 2.51 SD = 1.11	1	Death is only a passage. After their death, people continue to exist and remember this life’s experiences.	(1 = 5) (2 = 4) (3 = 3) (4 = 2) (5 = 1)	0.67
	2	Death is a definitive annihilation. After their death, people no longer exist, so they will not experience anything.	(1 = 1) (2 = 2) (3 = 3) (4 = 4) (5 = 5)	0.73
	3	Death is a definitive annihilation. After death, even if the others remember the person who is no longer there, she will no longer remember anything.	(1 = 1) (2 = 2) (3 = 3) (4 = 4) (5 = 5)	0.56
	4	Death is only a passage. After their death, people continue to exist and therefore to have new experiences.	(1 = 5) (2 = 4) (3 = 3) (4 = 2) (5 = 1)	0.67

^1^ In the analysis, only the students who completed all item of TDRS-C (*n* =209) are included.

**Table 3:** Goodness-of-Fit Indices of Confirmatory Factor Analysis models for Reflective Functioning Questionnaire (RFQ) ^1^ and Testoni Death Representation Scale for Children (TDRS-C) ^2^.

Model	N Item	Chi-Square	df	*p*	RMSEA	AIC Model	NNFI	CFI	GFI
RFQ									
One factor for RFQ_C	6	35.92	9	<0.001	0.12	3667.51	0.72	0.83	0.94
One factor for RFQ_U	6	2.95	9	0.966	0.00	3613.80	1.22	1.00	1.00
One factor for RFQ_Total	12	95.06	50	<0.001	0.07	7019.21	0.88	0.91	0.93
Two factors RFQ_C, RFQ_U	12	66.28	49	0.051	0.04	6992.42	0.95	0.97	0.95
TDRS-C									
One factor for TDRSC_Total	4	10.37	2	0.006	0.14	25.90.01	0.92	0.97	0.98

^1^ Only students who completed all items of RFQ (*n* = 205) are included in the analysis; ^2^ Only students who completed all items of TDRS-C (*n* = 209) are included in the analysis.

**Table 4:** Descriptive statistics of study variables by gender and age groups with ANOVA results (comparison group, *n* = 210).

Measures	9–10 Years				11–14 Years				Anova Results ^a^		
	Males		Females		Males		Females				
	M	DS	M	DS	M	DS	M	DS	Gender	Age	Gender × Age
RFQ_C	0.83	0.58	1.19	0.81	0.96	0.68	0.83	0.63	1.34	1.42	6.17 *
RFQ_U	0.85	0.55	0.85	0.72	0.68	0.46	0.92	0.59	1.97	0.45	1.93
TDRS-C	2.74	1.21	1.99	1.00	2.64	1.19	2.38	1.04	6.69 *	2.20	4.38 *
PA	59.73	7.66	58.77	11.35	54.28	9.06	53.58	11.02	2.15	21.41 ***	0.63
NA	27.21	9.10	27.46	10.07	26.77	8.52	32.53	12.33	7.33 **	5.13 *	6.49 *
SDQ_EMO	3.18	2.38	4.28	2.36	2.35	2.33	4.25	2.34	18.81 ***	1.63	1.30
SDQ_PEER	2.00	1.64	2.21	1.96	1.77	2.17	1.83	1.70	0.03	2.12	0.36
SDQ_Internalizing Problems	5.18	3.22	6.49	3.82	4.12	3.82	6.08	3.25	9.47 **	2.76	0.21
SDQ_COND	2.48	2.03	1.95	1.92	2.40	1.76	2.38	1.82	0.42	1.10	1.83
SDQ_HYPER	4.09	2.59	2.79	2.19	3.79	1.92	3.38	2.23	4.86 *	0.79	3.32
SDQ_Externalizing Problems	6.58	3.95	4.74	3.73	6.19	3.08	5.77	3.65	2.93	1.21	3.39
SDQ_Tot.	11.76	6.10	11.23	6.35	10.32	5.96	11.85	5.74	0.58	0.09	1.89
SDQ_PROS	7.33	1.59	7.62	2.12	7.18	1.65	7.90	1.70	5.49 *	0.29	1.41

^a^ Degrees of freedom are 1 and 206 for all F-test. * *p* < 0.05; ** *p* < 0.01; *** *p* < 0.001.

**Table 5:** Correlations between all measures (comparison group, *n* = 210).

Measures	1	2	3	4	5	6	7	8	9	10	11	12	13
1.RFQ_C													
2.RFQ_U	−0.55 ***												
3.TDRS	−0.06	0.07											
4.PA	−0.02	−0.05	−0.22 **										
5.NA	−0.23 **	0.30 ***	0.06	−0.43 ***									
6.SDQ_EMO	−0.20 **	0.35 ***	0.03	−0.27 ***	0.43 ***								
7.SDQ_PEER	−0.06	0.26 ***	−0.01	−0.24 ***	0.32 ***	0.30 ***							
8.Intern.Probl.	−0.18 **	0.38 ***	*0.01*	−0.32 ***	0.48 ***	0.87 ***	0.74 ***						
9.SDQ_COND	−0.36 ***	0.33 ***	0.17 *	−0.20 **	0.29 ***	0.26 ***	0.35 ***	0.37 ***					
10.SDQ_HYPER	−0.39 ***	0.29 ***	0.20 **	−0.31 ***	0.36 ***	0.23 **	0.30 ***	0.32 ***	0.53 ***				
11.Extern.Probl.	−0.43 ***	0.35 ***	0.21 **	−0.30 ***	0.38 ***	0.28 ***	0.37 ***	0.39 ***	0.85 ***	0.90 ***			
12.SDQ_Tot.	−0.37 ***	0.44 ***	0.14 *	−0.37 ***	0.51 ***	0.68 ***	0.66 ***	0.83 ***	0.73 ***	0.73 ***	0.84 ***		
13.SDQ_PROS	0.15 *	−0.07	−0.16 *	0.22 **	−0.07	0.07	−0.14 *	−0.02	−0.29 ***	−0.27 ***	−0.32 ***	−0.21 **	

* *p* < 0.05; ** *p* < 0.01; *** *p* < 0.001.

**Table 6:** Descriptive statistics of study variables for children of research and comparison group with *t*-test results and Cohen’s *d*.

Measures	Research Group (*n* = 38)		Comparison Group (*n* = 210)		*t* (246)	Cohen’s *d*
	M	DS	M	DS		
RFQ_C	0.80	0.76	0.93	0.68	−1.04	−0.18
RFQ_U	0.45	0.42	0.83	0.58	−3.88 ***	−0.68
TDRS	2.60	0.68	2.51	1.11	0.48	0.09
PA	52.87	12.07	54.73	10.24	−1.00	−0.18
NA	34.32	11.52	30.13	11.24	2.10 *	0.37
SDQ_EMO	3.84	2.88	3.57	2.48	0.61	0.11
SDQ_PEER	1.71	2.08	1.84	1.82	−0.40	−0.07
Intern.Probl.	5.55	4.60	5.41	3.49	0.22	0.04
SDQ_COND	2.45	1.88	2.40	1.83	0.13	0.02
SDQ_HYPER	4.55	2.65	3.60	2.22	2.35 *	0.41
Extern.Probl.	7.00	4.27	11.42	5.87	1.53	0.27
SDQ_Tot.	12.55	8.22	6.01	3.55	1.02	0.18
SDQ_PROS	8.00	1.68	7.61	1.69	1.29	0.23

* *p* < 0.05; *** *p* < 0.001.

## Data Availability

The datasets used during the current study are available from the corresponding author on reasonable request.
